# Estimation of chlorophyll content in cotton leaves using UAV RGB imagery

**DOI:** 10.3389/fpls.2026.1794351

**Published:** 2026-04-21

**Authors:** Yufen Huang, Zhenqi Fan, Weimo Wu, Hongxin Wu, Yanlong Liu, Qi Wang, Ximeng Zhang, Meiyun Li, Xianyin Duan, Ziqi Gao, Tianping Wang

**Affiliations:** 1 College of Information Engineering, Tarim University, Alaer, China; 2 Key Laboratory of Tarim Oasis Agriculture, Ministry of Education, Tarim University, Alaer, China; 3 College of Cyber Security, Tarim University, Alaer, China; 4 College of Agriculture, Tarim University, Alaer, China

**Keywords:** LCC, machine learning, precision agriculture, RTK, UAV RGB image

## Abstract

Accurate and non-destructive acquisition of leaf chlorophyll content (LCC) in cotton plant canopies is of significant importance for real-time monitoring of cotton growth and implementing precise water and nitrogen management in cotton fields. This study utilized UAV-based RGB imagery combined with real-time kinematic (RTK) technology to efficiently and accurately retrieve LCC under different nitrogen application levels in a cotton field, employing 6 machine learning algorithms: Least Absolute Shrinkage and Selection Operator regression (LASSO), Multiple Linear Regression (MLR), Partial Least Squares Regression (PLSR), Random Forest Regression (RFR), Ridge Regression (Ridge), and Support Vector Regression (SVR). Among these, the SVR model demonstrated the best overall performance, with coefficient of determination (R²), root mean square error (RMSE), relative root mean square error (rRMSE), and mean absolute percentage error (MAPE) values of 0.82, 0.14 mg/g, 8.91%, and 6.99% for the training set, and 0.75, 0.14 mg/g, 8.90%, and 7.83% for the testing set, respectively. Furthermore, the SVR model was applied to retrieve LCC pixel-by-pixel from UAV imagery, and pseudo-color rendering techniques were used to generate spatial distribution maps of LCC in the cotton canopy, visually presenting the spatial variability characteristics of LCC within the field. The results indicate that the cotton canopy LCC estimation method based on UAV RGB imagery combined with RTK technology achieves comparable accuracy to the more expensive multispectral and hyperspectral techniques, without a significant reduction in precision. This approach provides an efficient, low-cost, and reliable method for detecting canopy LCC in small-scale cotton fields.

## 1 Introduction

Chlorophyll plays a crucial role in the growth of cotton (Ustin et al., 2009; Jay et al., 2017; Cutolo et al., 2023; Kang et al., 2025a) and serves as a key indicator for characterizing photosynthetic capacity and growth status (Davies and Combrink, 1986; Steele et al., 2008; Chen and Chen, 2018). Since a significant proportion of nitrogen absorbed by cotton plants is utilized for the synthesis of chlorophyll molecules, changes in chlorophyll content can effectively reflect the nitrogen nutritional status of cotton plants (Filella et al., 1995; Restrepo-Diaz et al., 2016; Kang et al., 2025b), this establishes chlorophyll content as an important link connecting cotton metabolism with external nutrient supply (Roca et al., 2018; Liu et al., 2025).

Traditional methods for leaf chlorophyll content (LCC) determination mainly include spectrophotometry and SPAD chlorophyll meter measurements. Although spectrophotometry offers high accuracy, it requires leaf sample collection, laboratory extraction, and measurement—a process that is laborious, time-consuming, and destructive, making continuous dynamic monitoring of the same plant impossible (Zhang et al., 2023). The SPAD meter, while enabling non-destructive measurements, suffers from low manual operation efficiency and is difficult to apply at field scales to obtain sufficient samples for characterizing within-field spatial heterogeneity (Xu et al., 2026). These limitations of traditional methods pose significant challenges for real-time, large-scale, and high-resolution monitoring of cotton growth.

The pigments in leaves alter their reflective properties by absorbing specific bands of visible light, making optical vegetation indices constructed from reflectance in different spectral bands an effective means of estimating plant physiological parameters such as Leaf Area Index (LAI), Leaf Chlorophyll Content (LCC), and biomass (Gausman et al., 1969; Woolley, 1971). For instance, Bacour et al (Bacour et al., 2006). utilized neural networks to estimate LAI and canopy LCC using MERIS satellite reflectance data across 11 spectral bands. Verrelst et al. (2012). achieved accurate inversion of LCC by combining Gaussian process machine learning with hyperspectral data from CHRIS satellite’s 62 bands. Schlemmer et al. (2013) found that vegetation indices combining near-infrared with green or red-edge bands from Sentinel-2 satellite data can effectively retrieve chlorophyll and nitrogen content in maize canopies. However, satellite remote sensing is constrained by limitations in spatial and temporal resolution, making it difficult to meet the demands of fine-scale monitoring in small agricultural fields. In this context, unmanned aerial vehicle (UAV) remote sensing, with its flexible and efficient data acquisition capabilities, offers a new technological approach for monitoring crop growth status (Gong et al., 2018). Maresma et al. (2016) utilized UAV multispectral (MS) imagery, integrating vegetation indices, Soil and Plant Analyzer Development (SPAD) measurements, and plant height parameters to accurately predict maize yield. Saberioon and Gholizadeh (2016) developed novel vegetation indices suitable for chlorophyll monitoring by analyzing the influence of visible light bands in UAV imagery on rice plants. Wei et al. (2025) successfully achieved fine-scale monitoring of LCC in urban trees by leveraging the ultra-high spectral resolution of hyperspectral (HS) UAVs. Notably, Yuan et al. (2022) conducted a comparative study on the effectiveness of RGB and MS UAVs in monitoring the SPAD values of Cephalotaxus hainanensis under shaded conditions. Their results indicated that vegetation indices based on MS data (R² = 0.938) significantly outperformed those based on RGB indices (R² < 0.8) in terms of retrieval accuracy.

Although multispectral (MS) and hyperspectral (HS) UAVs demonstrate clear advantages in chlorophyll retrieval accuracy, their high equipment costs and complex operational requirements limit their widespread adoption among ordinary farmers. In contrast, RGB UAVs, with their lower economic cost, user-friendly operation, and high spatial resolution, offer unique advantages for promoting the adoption of precision field management technologies. For instance, Yi et al. (2025). achieved a monitoring accuracy of over 75% for winter wheat and rye biomass under nitrogen-deficient conditions using more than 3,600 RGB images. Similarly, Tripathi et al. (2025). attained high-precision prediction of rice yield (RMSE = 0.27 t ha^−1^) by combining UAV RGB imagery with a random forest model. Additionally, Guo Y. et al. (2020). successfully established a linear relationship between RGB-based vegetation indices and maize SPAD values using UAV RGB images integrated with machine learning, achieving a peak R² of 0.85.

In summary, while UAV RGB imagery may not match the spectral richness of MS and HS systems, it remains capable of providing reliable decision support for field management. However, existing studies on chlorophyll retrieval based on UAV RGB predominantly rely on aggregated statistical features of canopy imagery. A critical limitation lies in the general lack of high-precision spatial alignment between ground-measured leaf samples and UAV image pixels. This “positional ambiguity” significantly constrains the retrieval accuracy of models and the reliability of spatial visualization. To address this core issue, the key innovation of this study lies in the systematic integration of Real-Time Kinematic (RTK) technology into the leaf sample geolocation process, achieving, for the first time, centimeter-level precise registration between leaf sample positions and UAV image pixels. This technical approach fundamentally ensures, at the data source, that the image features used for modeling accurately represent the biochemical parameters of the corresponding leaves, thereby minimizing errors introduced by positional deviations and laying a solid data foundation for high-precision inversion. Building upon this foundation, this study systematically investigated UAV RGB imagery-based methods for estimating cotton canopy LCC. By comparing six machine learning algorithms, screening key color features, and optimizing model parameters, we established an LCC inversion framework that integrates both high accuracy and low cost. This framework not only provides a feasible solution to the challenge of spatial matching between ground samples and remote sensing imagery but also offers technical support for promoting the practical application of low-cost RGB UAVs in small-scale cotton field precision fertilization management, facilitating the transition of precision agriculture technologies from the laboratory to the field and enabling their democratized and large-scale adoption. The generated LCC spatial distribution maps intuitively reveal within-field spatial variability of nitrogen nutrition, enabling agricultural managers to rapidly identify “nitrogen-deficient” and “nitrogen-rich” areas. These maps can be used to develop variable-rate fertilization prescription maps, guiding fertilization machinery to apply different nitrogen rates across different zones, thereby achieving “on-demand fertilization” and avoiding the fertilizer waste and environmental pollution associated with traditional uniform fertilization practices.

## 2 Materials and methods

### 2.1 Experimental design

**Figure 1:**
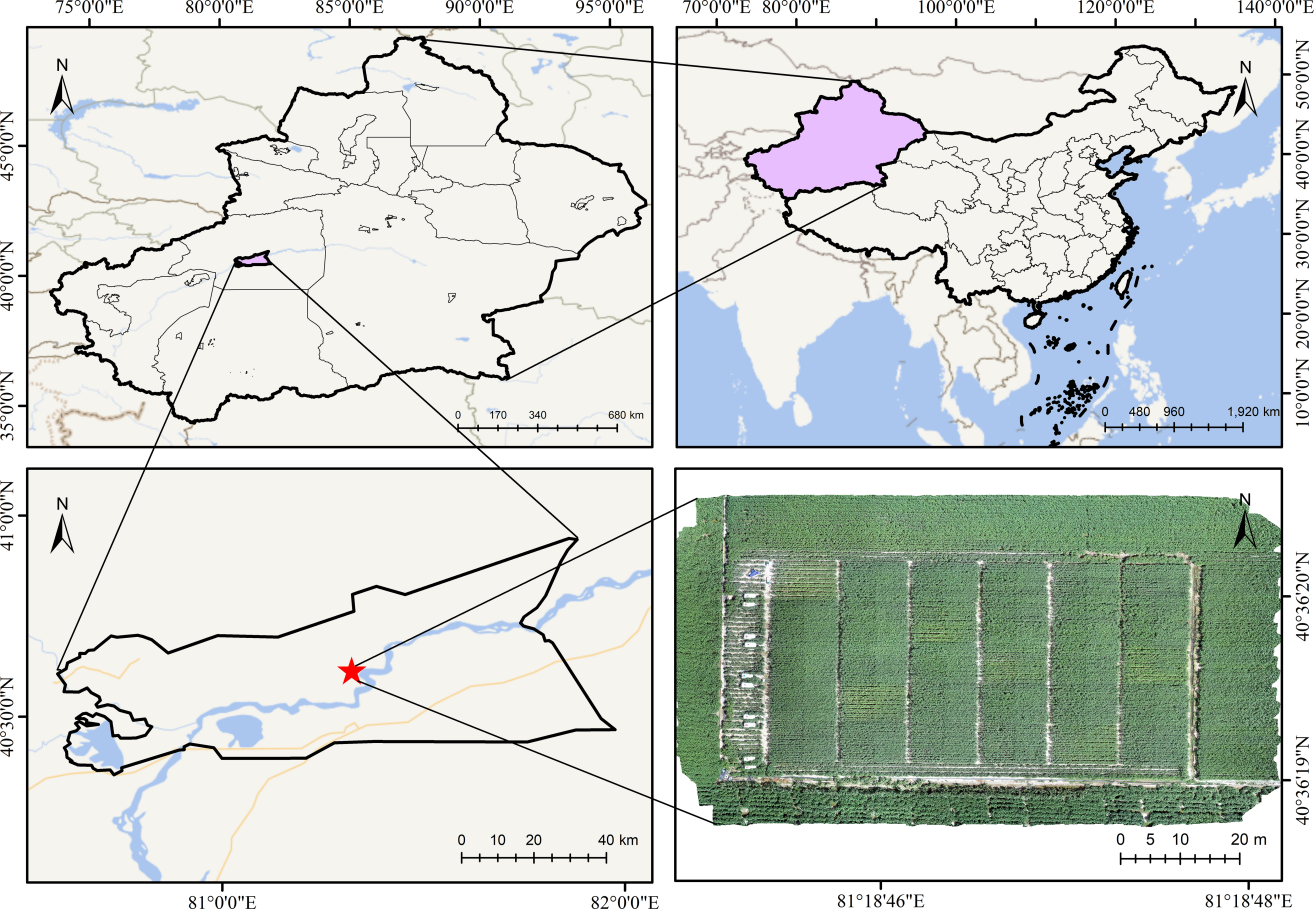
Overview map of the experimental area.

This study was conducted at the Alaer Experimental Station of the Cotton Research Institute, Chinese Academy of Agricultural Sciences (Tenth Regiment, Alaer City, First Division, Xinjiang Production and Construction Corps; 40°36′37.6″N, 81°19′43.3″E). An overview of the experimental area is shown in **Figure 1**. The soil in the experimental region is characterized by low organic matter content and generally falls into the category of lightly saline soil (Li et al., 2023), which is typical of the soil types in the Alar reclamation area.

**Figure 2:**
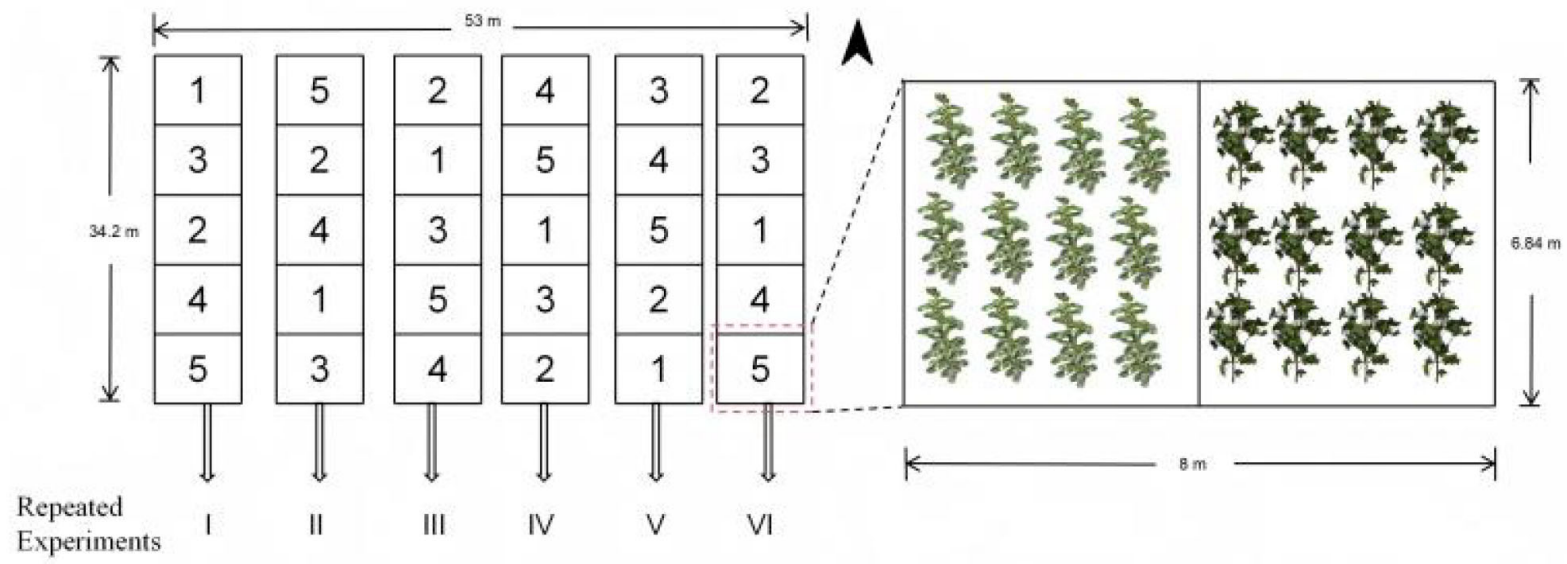
Overview of planting in the experimental site.

On April 15, 2024, after mechanical film mulching, manual dibbling was carried out using an ultra-wide film (width 2.05 m) planting pattern with one film accommodating 6 rows of cotton and three drip irrigation tapes, designed for mechanical harvesting. The planting rows were oriented east-west, with row spacing arranged as 10 cm - 66 cm - 10 cm - 66 cm - 10 cm, and plant spacing within rows set at 10 cm. The specific layout of the plots is illustrated in **Figure 2**. Fertilizer application was managed using a self-developed integrated water-fertilizer system, with drip irrigation and fertilization conducted independently for each plot. The irrigation volume was consistent across all plots, with a total of 10 drip irrigation events throughout the growth period, amounting to 4950 m³/ha of irrigation water.

The cotton varieties involved in the experiment were Tahe 2 and Xinhai 21, which are the primary cultivars grown in the Alaer reclamation area. The former is an upland cotton variety suitable for early to mid-maturing cotton regions in southern Xinjiang, while the latter has been the dominant sea island cotton variety in the Aksu region of Xinjiang since the 1990s (Mariani and Ferrante, 2017; Wang et al., 2022). In terms of plant architecture, Tahe 2 exhibits a more compact canopy and shorter stature compared to Xinhai 21. This study adopted a single-factor completely randomized block design, referencing the Chinese agricultural industry standard NY/T 1536–2007 Technical Specification for Field Trials of Microbial Fertilizers and Guidelines for Fertilizer Efficiency Evaluation. The experiment comprised 5 water-fertilizer treatments, as detailed in **Table 1**.

**Table 1:** Experimental design.

Treatment	Treatment details
1	Blank control (no fertilizer application)
2	Standard control (conventional nitrogen application rate: 400 kg N/ha)
3	10% nitrogen reduction (360 kg N/ha)
4	10% nitrogen reduction combined with chitosan oligosaccharide (degree of polymerization 2-6, denoted as Chitosan Oligosaccharide 1, molecular weight ≤ 2,000, content ≥ 80%)
5	10% nitrogen reduction combined with chitosan oligosaccharide (degree of polymerization 2-20, denoted as Chitosan Oligosaccharide 2, molecular weight ≤ 2,000, content ≥ 90%)

In treatments 4 and 5, chitosan oligosaccharide 1 and chitosan oligosaccharide 2 were sourced from manufacturers in Dalian and Qingdao, respectively. To promote plant growth, enhance photosynthetic efficiency, and improve crop stress resistance (Ou et al., 2022; Kerch et al., 2011; Sajid et al., 2020), chitosan oligosaccharides were diluted at specific ratios and applied as foliar sprays in this study. The application rate for chitosan oligosaccharides was 450 g/ha per treatment, with a total of eight spray applications throughout the experiment. The total amounts of phosphorus and potassium fertilizers were consistent across all treatments, applied at rates of 140 kg/ha and 130 kg/ha, respectively. Each treatment was replicated 6 times, resulting in a total of 30 plots. Each plot measured 8 m in length and 6.84 m in width. Within each plot, Xinhai 21 was planted on the western 4 m side, and Tahe 2 on the eastern 4 m side. The treatments were arranged following a randomized complete block design. The distribution of experimental plots is illustrated in **Figure 2**.

### 2.2 Collection and processing of cotton canopy images

**Figure 3:**
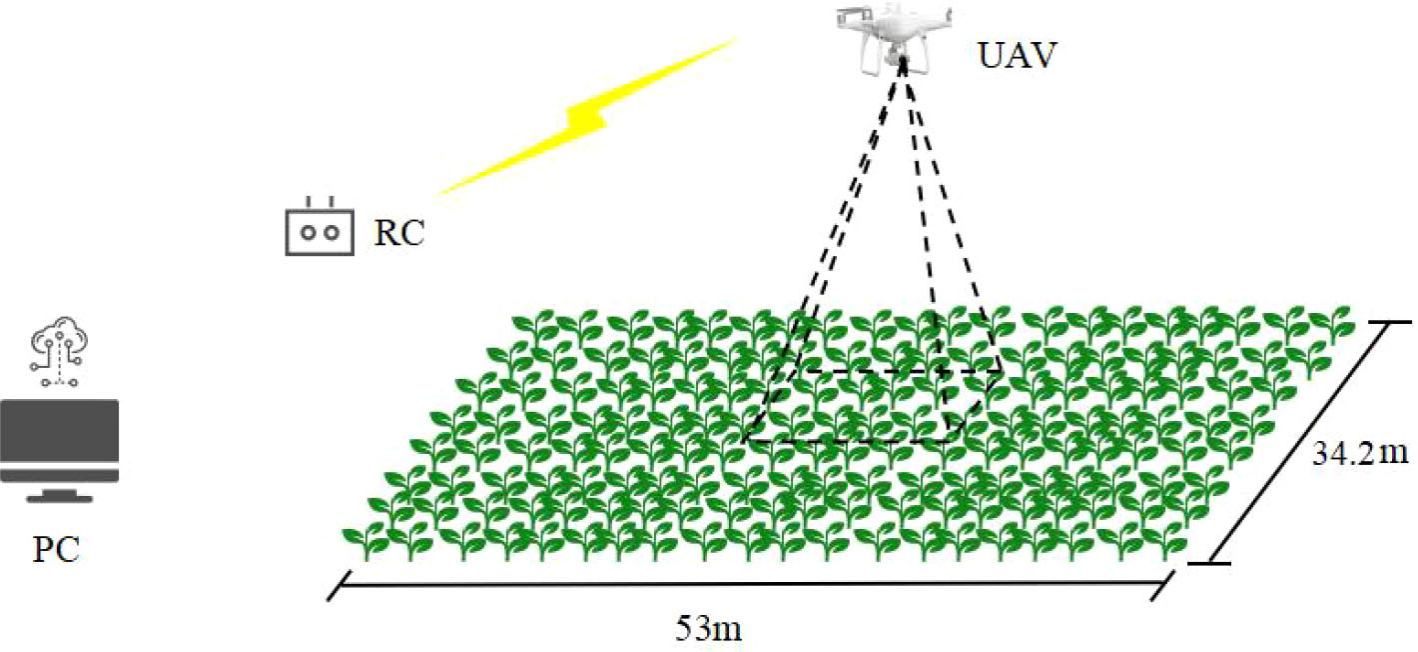
UAV information acquisition.

On July 12, 2024, cotton leaf sampling during the bud stage and UAV data acquisition were carried out. To obtain high-precision cotton canopy image data, this study employed the DJI Phantom 4 RTK imaging system for aerial remote sensing operations. The UAV remote sensing platform primarily consisted of the DJI Phantom 4 RTK MSDK (screenless version) GL300L aircraft, the FC6310R high-resolution camera, and a ground data processing terminal. The camera was equipped with a 1-inch CMOS image sensor, offering an effective pixel count of 20 million (total pixel count: 20.48 million). The UAV information acquisition setup is illustrated in **Figure 3**.

To minimize the impact of environmental factors on image quality, key environmental conditions were strictly controlled during data acquisition. Flights were conducted under clear, cloudless skies with stable illumination conditions between 12:00-14:00 local time. Flight operations were performed only when wind speed was below 3 m/s to avoid image blurring and geometric distortion. Image acquisition for all plots was completed within one consecutive hour to ensure consistent illumination conditions across the entire study area.

During the image acquisition phase, systematic data collection was conducted using the mapping aerial photography mode. Key flight parameters were set as follows: flight speed at 3 m/s and relative flight altitude at 13 m. By optimizing the flight route planning and overlap design (with a longitudinal overlap of 80% and a lateral overlap of 70%), a total of 377 valid canopy images were captured.

**Figure 4:**
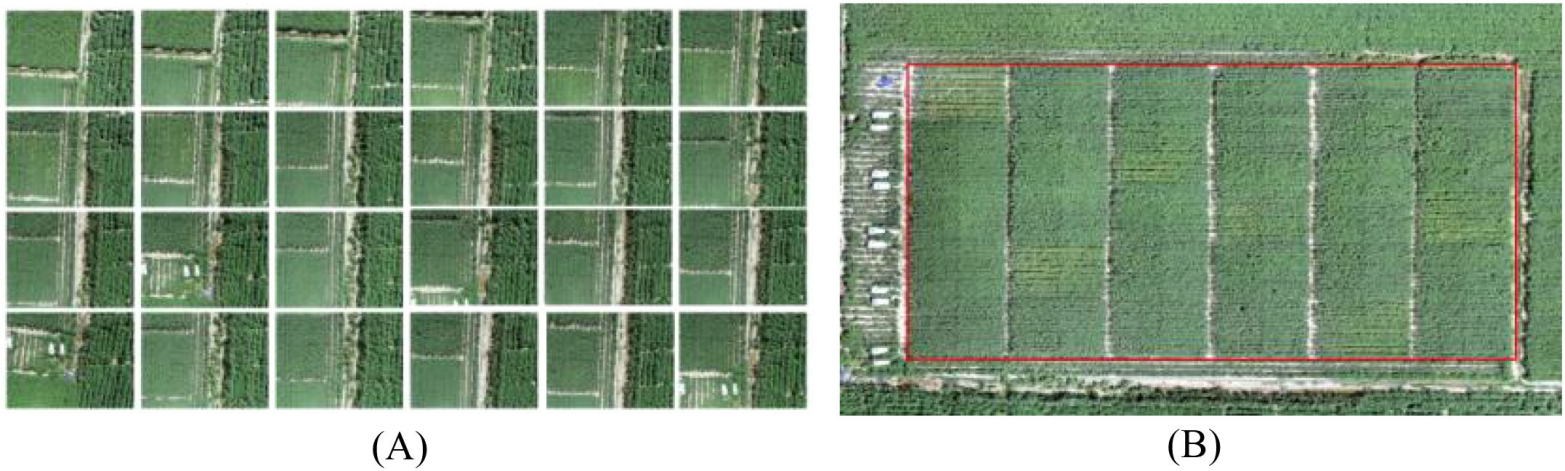
**(A)** Single image; **(B)** Stitched image.

The images were stitched using Pix4Dmapper 4.4.12 software through a series of steps including feature point matching, aerial triangulation densification, and digital surface model generation, resulting in the creation of a high spatial resolution orthomosaic. The stitched orthomosaic had a pixel size of 3.82 × 10^−8^ degrees in both the X and Y directions, indicating square pixels. Based on the latitude of the study area (approximately 40.6°N), this corresponds to a ground sampling distance (GSD) of approximately 0.4 cm/pixel. The final stitched cotton canopy image is shown in **Figure 4**, where (A) represents a single image captured by the UAV, and (B) displays the global stitched image of the experimental area. The red-marked section in **Figure 4B** indicates the specific experimental plot under study in this research.

**Figure 5:**
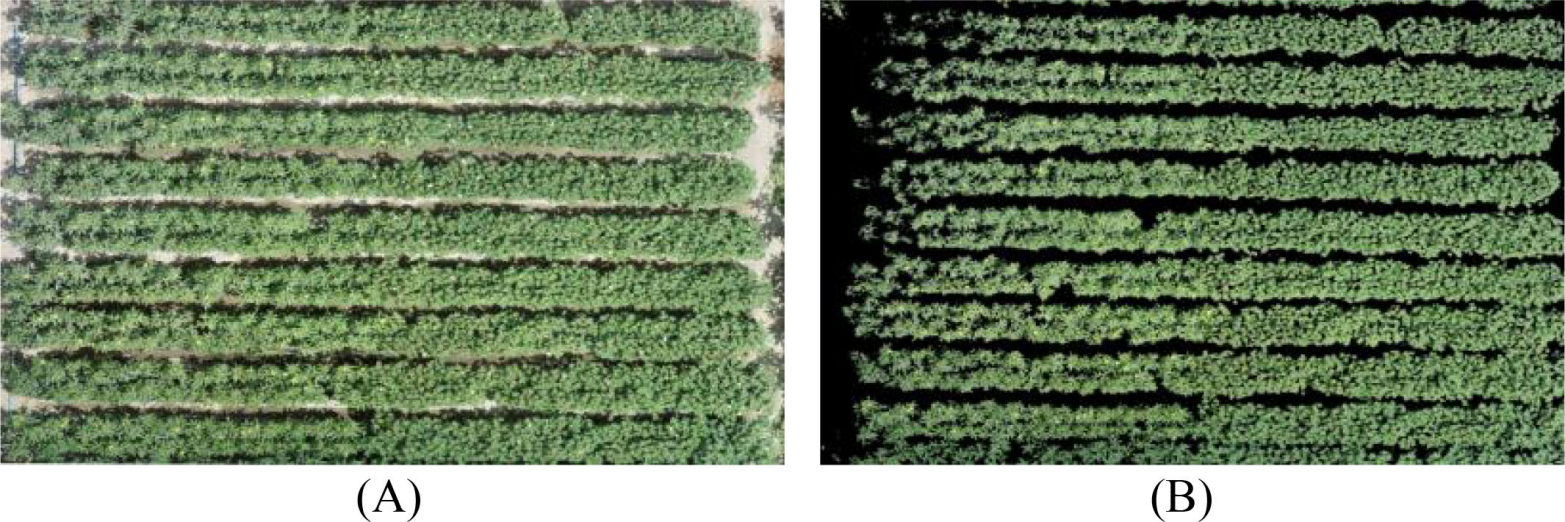
**(A)** original image; **(B)** segmentation results based on CIVE and Otsu.

Since the Color Index of Vegetation Extraction (CIVE) enhances the color contrast between vegetation and soil by calculating specific band combinations, it effectively highlights vegetation information (Tao and Jin, 2023; Sun et al., 2024). The Maximum inter-class variance method (Otsu) can automatically identify an optimal threshold to maximize the variance between foreground and background classes for accurate binarization segmentation (Xu et al., 2011; Guo et al., 2014). Therefore, to distinguish soil from vegetation, the Otsu threshold segmentation technique was applied to cotton canopy images based on CIVE color features (calculation formula provided in **Table 2**), achieving separation between crops and soil. Shadow areas were identified using a combination of brightness threshold (digital number < 50) and CIVE index values, and were excluded as non-vegetation pixels along with bare soil, field roads, and other non-vegetation features. **Figure 5A** shows the original image, while **Figure 5B** presents the segmentation results based on CIVE and Otsu.

**Table 2:** 10 color features.

Name	Parameter/formula	Reference
Ratio Yellowness Index (RYI)	G/B	Shao, C (Shao et al., 2023).
Green Leaf Index (GLI)	((G-R)+(G-B))/(2*G+R+B)	Louhaichi (Louhaichi et al., 2001)
Excess Green Excess Red Difference Index (EXGR)	EXG -EXR	George E. Meyer (Meyer and Neto, 2008)
Normalized Difference Index (NDI)	(G-R)/(G+R)	Hunt, E.R (Hunt et al., 2005).
Normalized Difference Yellowness Index (NDYI)	(G-B)/(G+B)	Woebbecke (David et al., 1993)
Excess Red Parameter (EXR)	1.4r -g	George E. Meyer (Woebbecke et al., 1994)
Excess Green Parameter (EXG)	2g-r-b	Woebbecke (David et al., 1993)
Red-Green-Blue Vegetation Index (RGBVI)	(G^2-R*B)/(G^2+RB)	Juliane Bendig (Bendig et al., 2015)
Color Index of Vegetation Extraction (CIVE)	0.441r-0.811g+0.385b+18.48745	M. Guijarro (Guijarro et al., 2011)
Green-Red Ratio Index (GRI)	G/R	Athos Agapiou (2020)

(Note: In the formulas, r, g, b represent normalized red, green, and blue components, i.e., r = R/(R+G+B), g = G/(R+G+B), b = B/(R+G+B)).

### 2.3 Collection and processing of cotton leaf samples

Two cotton varieties, ‘Xinhai 21’ and ‘Tahe 2’, were planted on the eastern and western sides of each plot, respectively. During sampling, a 1.5 m buffer zone was set on both the length and width sides of each plot. Ten sampling points were randomly selected within the planting area of each variety, and one fully expanded functional leaf (the 3rd to 4th leaf from the main stem apex, avoiding leaf veins and diseased or damaged leaves) was collected from each sampling point. The ten leaves from the same variety were pooled and homogenized to form one composite sample, representing the average leaf chlorophyll content level of that variety in that plot. Using this method, a total of 60 composite samples were obtained from the 30 plots (30 plots × 2 varieties). Each collected leaf was precisely georeferenced at its central point using a Lite-RTK (WHand) device (manufactured by Wuhan Huancan Engineering Technology Co., Ltd.), which recorded the latitude, longitude, and elevation of the leaf samples. This instrument employs RTK technology, which achieves a static planar positioning accuracy of ±(3.0 + 0.5 × 10^−6^ D) mm and an elevation accuracy of ±(5 + 0.5 × 10^−6^ D) mm. In RTK mode, it maintains a planar accuracy of ±(10 + 1 × 10^−6^ D) mm and an elevation accuracy of ±(15 + 1 × 10^−6^ D) mm, where D represents the distance between measured points. During field operations, the calibrated RTK device was gently positioned above the target leaf for data collection. After satellite signal acquisition at the sampling point, the system performed real-time carrier phase differential processing to resolve three-dimensional coordinates with centimeter-level accuracy. This procedure provides a reliable geometric foundation for subsequent pixel-level registration between leaf samples and UAV imagery.

After completing the collection of geographic coordinate data, the functional cotton leaves were manually picked, tightly wrapped in aluminum foil, and immediately frozen in liquid nitrogen to preserve their biological activity and chemical stability. All collected samples were subsequently transported to the laboratory for chlorophyll extraction and measurement. This study accumulated a total of 60 chlorophyll samples for testing and 300 high-precision geographical data points.

The DJI Phantom 4 RTK UAV, used for image acquisition in this study, is equipped with a high-precision GNSS module. It employs real-time differential positioning technology, achieving a horizontal positioning accuracy of ±1 cm + 1 ppm (RMS, Root Mean Square) and a vertical positioning accuracy of ±1.5 cm + 1 ppm (RMS). Here, 1 ppm signifies an error increase of 1 mm for every kilometer the UAV moves.

**Figure 6:**
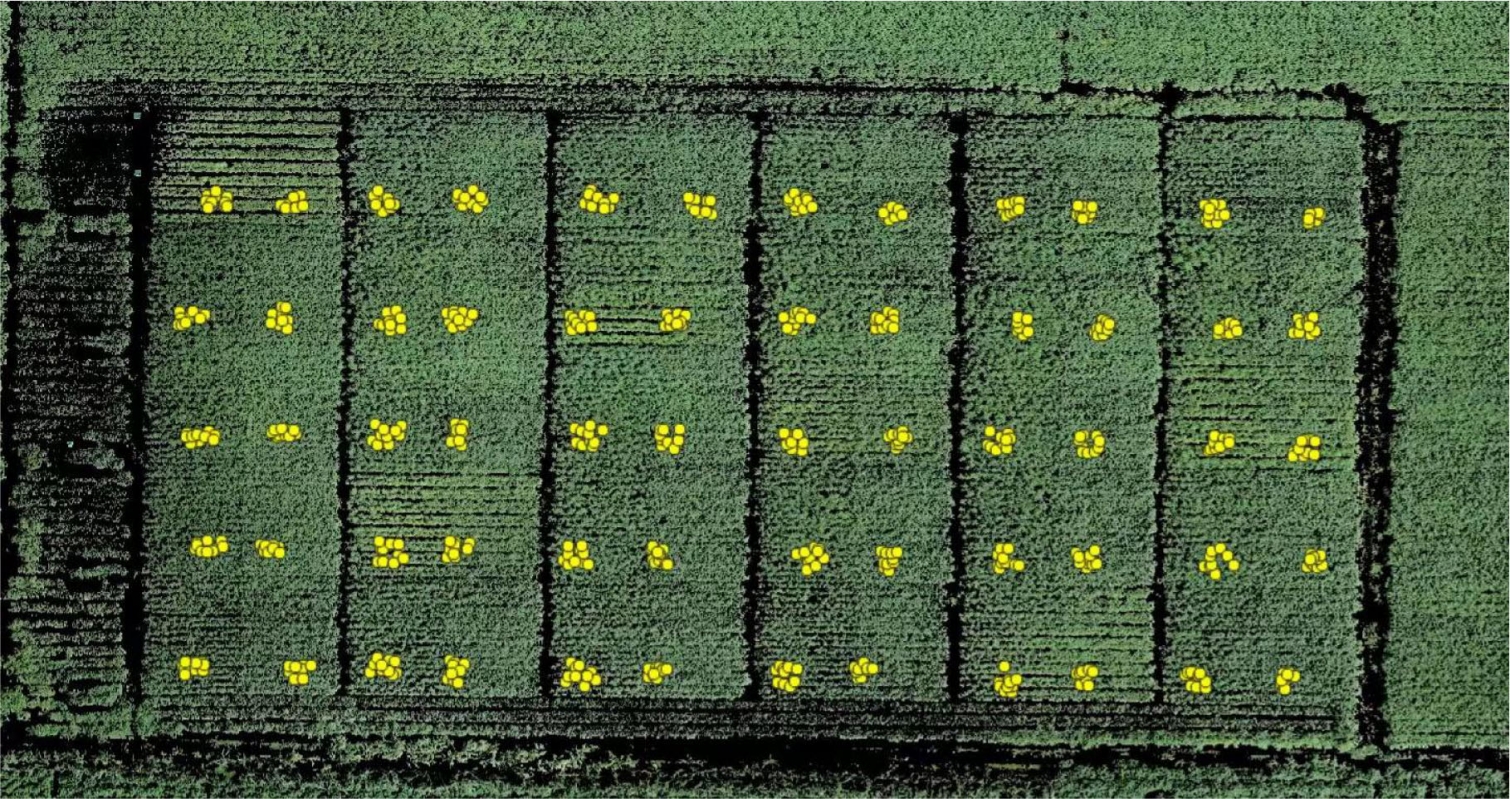
Matching sample points with global images.

The stitched cotton canopy raster image was imported into QGIS Desktop 3.40.6, and the information table containing leaf sample points was also loaded into the software. Using the “Create Points Layer from Table” function, the point data from the table was matched with the canopy image, thereby achieving geographical information alignment and spatial registration of the sample points. A circular buffer with a radius of 2.5 cm was generated around each registered leaf sample point as the region of interest (ROI). This radius was determined based on the average size of cotton functional leaves. Under the condition of GSD = 0.4 cm/pixel, each ROI contained approximately 120 pixels. The results of the geographical information matching for the sample points are shown in **Figure 6**.

### 2.4 Chlorophyll content determination

For chlorophyll content determination, the five leaves collected from each variety within a given plot were pooled, crushed, and thoroughly mixed to form a composite sample. This resulted in two composite samples per plot (one per variety). Chlorophyll content was measured following the spectrophotometric method specified in the national standard NY/T 3082-2017. An aliquot of 0.5 g was accurately weighed from the composite sample and placed into a conical flask, followed by the addition of 10 mL of a 1:1 mixture of anhydrous ethanol and acetone (hereinafter referred to as the extractant). The flask was sealed with sealing film and kept at room temperature in dark conditions for 5 hours to facilitate extraction. The solution was then filtered. A blank control was prepared using pure extraction solvent, and the instrument reading was zeroed accordingly. The absorbance of the test solution was measured at wavelengths of 645 nm and 663 nm, respectively.

The contents of chlorophyll a, chlorophyll b, and total chlorophyll in the sample, expressed as mass fractions and denoted as $\omega_{1}$, $\omega_{2}$ and $\omega_{3}$, were calculated using Equations 1–3, respectively.

(1) $${\text{ω}}_{1}=(12.72\times A_{1}-2.59\times A_{2})\times \text{ν/}(1000\times \text{m})$$

(2) $${\text{ω}}_{2}=(22.88\times A_{2}-4.67\times A_{1})\times v/(1000\times \text{m})$$

(3) $${\text{ω}}_{3}=(8.05\times A_{1}+20.29\times A_{2})\times v/(1000\times \text{m})$$

Formula Variables and Units:

$\omega_{1}:\text{ Chlorophyll a content (mg/g)}$

$\omega_{2}:\text{ Chlorophyll b content (mg/g)}$

$\omega_{3}:\text{ Total chlorophyll content (mg/g)}$

A1: Absorbance of the test solution at 663 nm

A2: Absorbance of the test solution at 645 nm

v: Volume of the test solution (mL)

m: Mass of the sample (g)

Calculation results should retain three significant figures.

Higher $\omega_{3}$ generally indicates sufficient nitrogen nutrition and vigorous overall growth (Zhang et al., 2022; Zhao et al., 2025). Therefore, inverting $\omega_{3}$ rather than measuring individual components, can more comprehensively reflect the real-time growth status and nutritional level of cotton plants. Statistical analysis of LCC data from 60 cotton leaf samples revealed a range of 0.65–3.10 mg/g, with a mean of 1.60 mg/g and a standard deviation of 0.46 mg/g. To generate a continuous and smooth spatial distribution map of cotton canopy LCC, Empirical Bayesian Kriging (EBK) in the ArcGIS 10.8 platform was employed to interpolate the 60 original samples. EBK is a geostatistical interpolation method that automatically estimates semivariogram model parameters. Compared to traditional kriging methods, it simulates multiple semivariogram models and accounts for their uncertainty, making it particularly suitable for non-stationary and non-Gaussian spatial data (Pilz and Spöck, 2008; Allard, 2013; Krivoruchko and Gribov, 2014; Krivoruchko and Gribov, 2019). The EBK parameters were set as follows: the semivariogram model was the Power model, with a subset size of 100, an overlap factor of 1, and 100 simulations. Empirical Bayesian log transformation was applied, and a prediction surface was generated. After generating the continuous interpolation surface, it was classified into 10 classes using the Geometrical Interval method. Within each class, nine new sample points were extracted using systematic random sampling (equal-interval sampling after sorting by pixel values), resulting in a total of 90 new points. The LCC values for the new points were taken from the predicted values of the interpolation surface.

From the results, the expanded sample dataset maintained the original value range (0.65–3.10 mg/g), with a mean value (1.61 mg/g) highly consistent with the original data. The standard deviation decreased from 0.46 mg/g to 0.32 mg/g, indicating that this method effectively preserved the overall distribution characteristics of the data while smoothing out potential random variations caused by the limited sample size. This adjustment better aligns the dataset with the actual spatial distribution patterns of chlorophyll content in small-scale cotton fields.

The obtained 150 sample data were randomly divided into a calibration set (105 samples) and a validation set (45 samples) at a ratio of 7:3. The specific values are presented in **Table 3**.

**Table 3:** Sample chlorophyll content statistics.

Sample type	Sample number	LCC (mg/g)							
		Maximum value	Minimum value	Mean value	Standard deviation	Coefficient of variation (%)	Quartile		
							25%	50%	75%
Original Sample	60	3.10	0.65	1.60	0.46	28.72	1.30	1.54	1.82
Sample after interpolation method	150	3.10	0.65	1.61	0.32	19.87	1.46	1.57	1.78

### 2.5 Selection of color feature index

The UAV images collected in the experiment are in RGB color space, with the R, G, and B components corresponding to the 622–760 nm, 492–577 nm, and 435–450 nm spectral bands, respectively. Within each ROI, only pixels identified as valid by the vegetation mask were used. The mean digital number values of the red, green, and blue bands were calculated as the spectral features for each sample. The mean was adopted instead of single-pixel values to effectively reduce random noise and represent the overall spectral response characteristics of the leaf. Based on the typical spectral characteristics of chlorophyll a and b, which exhibit absorption valleys in the 435–450 nm and 622–760 nm bands and a reflection peak in the 492–577 nm band, the RGB components and their combinations as color features can be utilized for monitoring cotton chlorophyll content. Color feature indices, as a category of empirical indicators that effectively reflect surface vegetation conditions, are characterized by simplicity and efficiency. Hunt et al. (2011) demonstrated that incorporating color features from remote sensing images enables more accurate characterization of vegetation health, and such features have become significant input variables for retrieving vegetation parameters. Consequently, this study evaluated the correlation between LCC and 10 color features (as listed in **Table 2**) using Pearson correlation analysis. The aim was to eliminate weakly correlated or interfering color features and select those with high correlation to LCC as input variables for the inversion model.

**Figure 7:**
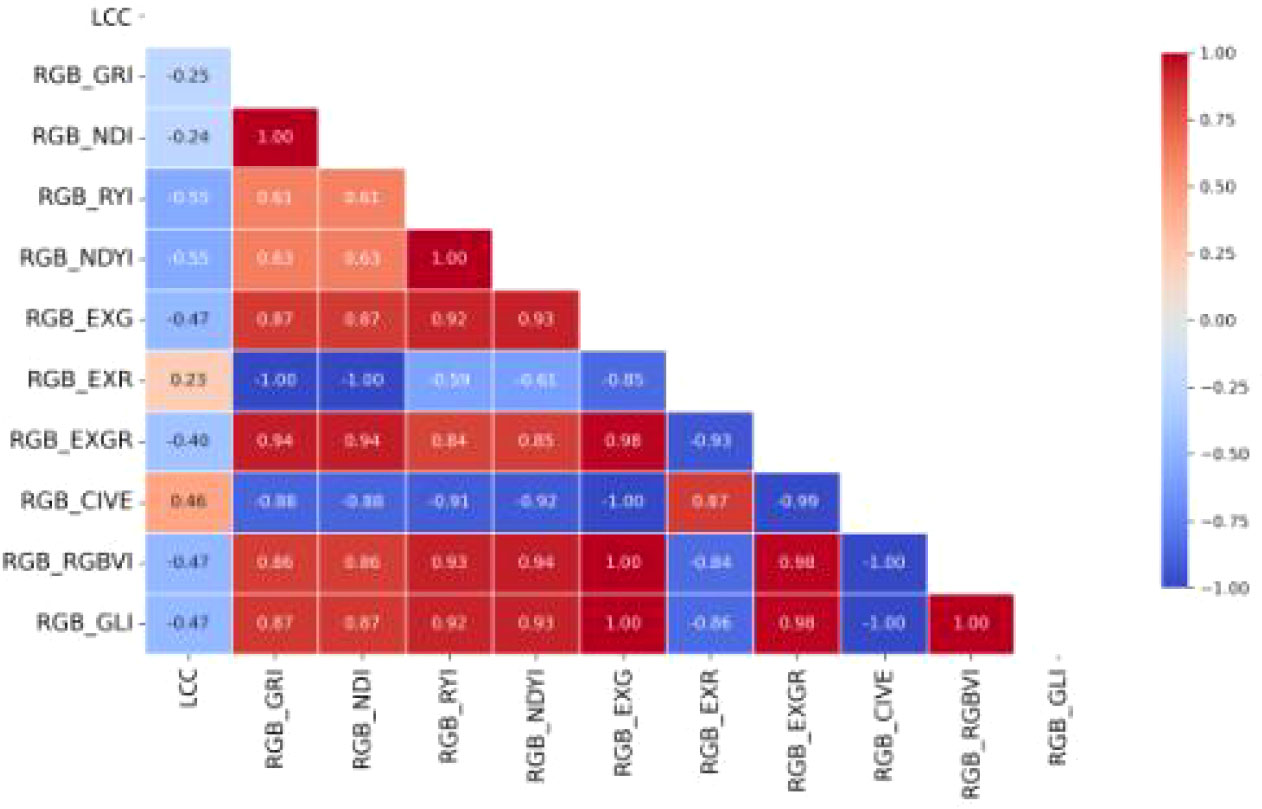
Color features and LCC correlation heatmap.

As shown in **Figure 7**, the color features exhibiting relatively high correlation coefficients with LCC include RYI, NDYI, EXG, CIVE, RGBVI, and GLI, totaling 6 features. Among these, CIVE demonstrates a strong positive correlation, while the other 5 features show strong negative correlations.

### 2.6 Model construction and accuracy evaluation

This study utilized six color feature values extracted from UAV RGB imagery, as selected in Section 2.5, to construct cotton LCC inversion models using six machine learning algorithms: LASSO (Tibshirani, 1996; Wang et al., 2019; Berk et al., 2024; Simon et al., 2024) (Least Absolute Shrinkage and Selection Operator), MLR (Hammond et al., 2023; Oliveira et al., 2012) (Multiple Linear Regression), PLSR (Guo A. et al., 2021; Maimaitijiang et al., 2019; Pan et al., 2023) (Partial Least Squares Regression), RFR (Kumar et al., 2023; Zhao et al., 2022) (Random Forest Regression), Ridge Regression (Chen et al., 2018; Shalabh, 2022), and SVR (Siripatrawan and Makino, 2024; Zhang et al., 2024) (Support Vector Regression). The evaluation criteria for the cotton LCC inversion models in this study included the coefficient of determination (R²), root mean square error (RMSE), relative root mean square error (rRMSE), and mean absolute percentage error (MAPE). A higher R² value indicates better model fit to the data, while lower values of RMSE, rRMSE, and MAPE denote higher model accuracy. The calculation formulas are provided in **Table 4**.

**Table 4:** Evaluation criteria formula table.

No.	Formula
1	$R^{2}=1-\frac{\sum_{i=1}^{n}{\left(y_{\text{i}}-{\hat{y}}_{i}\right)}^{2}}{\sum_{i=1}^{n}{\left(y_{i}-\bar{y}\right)}^{2}}$
2	$RMSE=\sqrt{\frac{1}{n}\sum_{i=1}^{n}{\left(y_{\text{i}}-{\hat{y}}_{i}\right)}^{2}}$
3	$rRMSE=\sqrt{\frac{\sum_{i=1}^{n}{\left(y_{i}-{\hat{y}}_{i}\right)}^{2}}{\sum_{i=1}^{n}y_{i}^{2}}}\times 100\%$
4	$MAPE=\frac{1}{n}\sum_{i=1}^{n}\left|\frac{y_{i}-{\hat{y}}_{i}}{y_{i}}\right|\times 100\%$

In the formulas: yi represents the measured chlorophyll content of the i-th sample; ${\hat{\text{y}}}_{\text{i}}$ represents the predicted chlorophyll content of the i-th sample; $\bar{\text{y}}$ represents the mean of the measured chlorophyll content values; n denotes the sample size.

To ensure fair comparison of all machine learning models and avoid performance bias caused by improper default parameter settings, hyperparameter tuning for all models was performed using grid search combined with 5-fold cross-validation. The tuning process was conducted based on the training set, with the optimal hyperparameters selected by minimizing the average RMSE of cross-validation. The search ranges and the corresponding optimal values for each model are presented in **Table 5**.

**Table 5:** Hyperparameter search ranges and optimal values for each machine learning model.

Model	Hyperparameter	Search scope	Optimal value
LASSO	alpha	[0.001, 0.01, 0.1, 1, 10]	0.01
MLR	-	-	-
PLSR	n_components	[2, 3, 4, 5, 6]	3
RFR	n_estimators	[50, 100, 200]	100
	max_depth	[5, 10, 20, None]	10
	min_samples_split	[2, 5, 10]	5
	min_samples_leaf	[1, 2, 4]	2
Ridge	alpha	[0.1, 1.0, 10.0]	1.0
SVR	kernel	[‘linear’, ‘poly’, ‘rbf’, ‘sigmoid’]	‘rbf’
	C	[0.1, 1, 10, 100]	10
	gamma	[‘scale’, ‘auto’, 0.01, 0.1, 1]	‘scale’
	epsilon	[0.01, 0.1, 0.2, 0.5]	0.1

MLR (Multiple Linear Regression) has no hyperparameters to tune. The random_state for RFR was fixed at 42 to ensure reproducibility. For SVR, gamma = ‘scale’ means gamma = 1/(n_features × X.var()).

**Figure 8:**
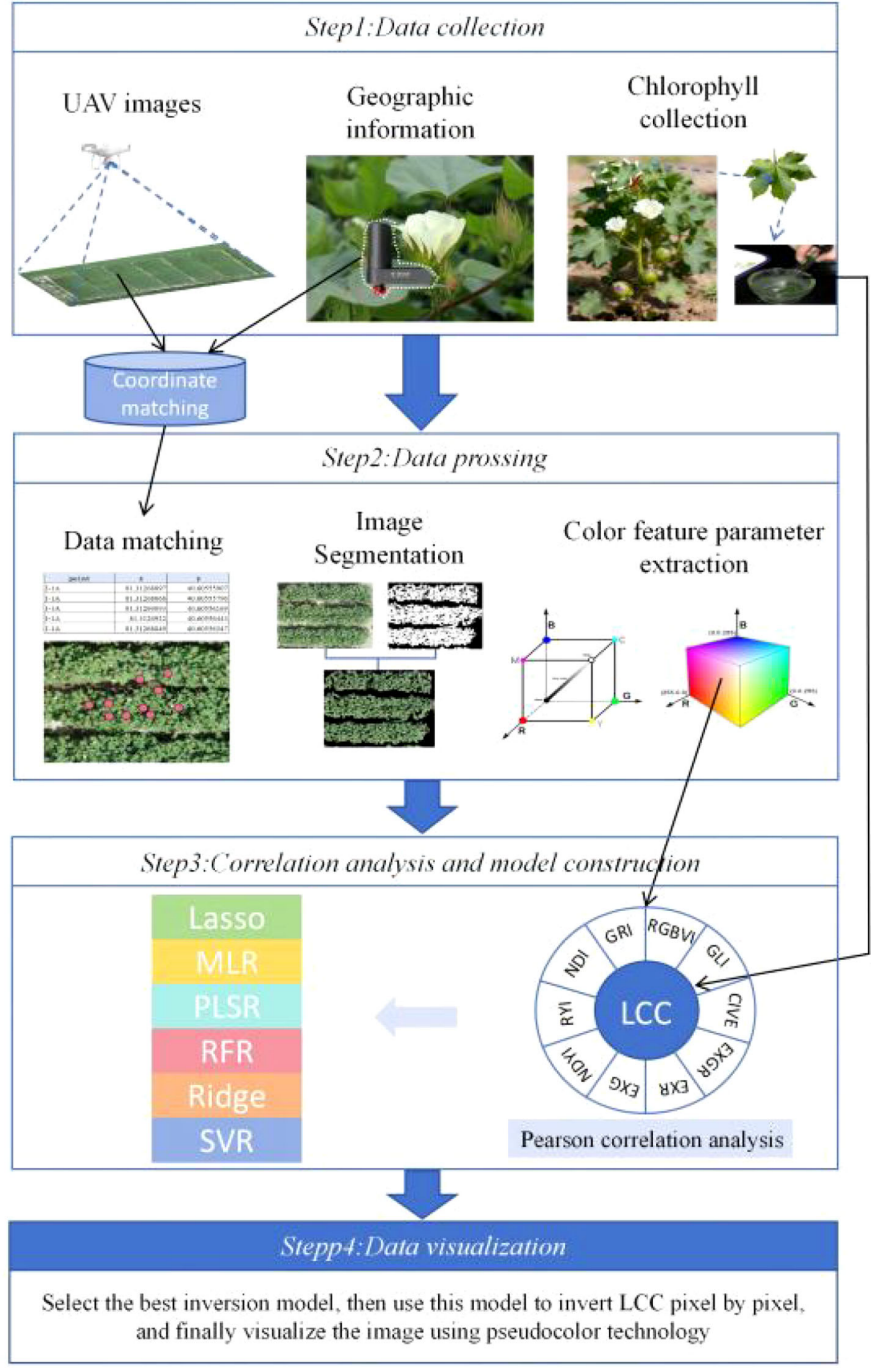
Technical workflow diagram.

The technical workflow of this study is illustrated in **Figure 8**, which is divided into four main parts: Part 1: Data Acquisition, Part 2: Data Processing, Part 3: Correlation Analysis and Model Construction, and Part 4: Data Visualization.

## 3 Results

**Figure 9:**
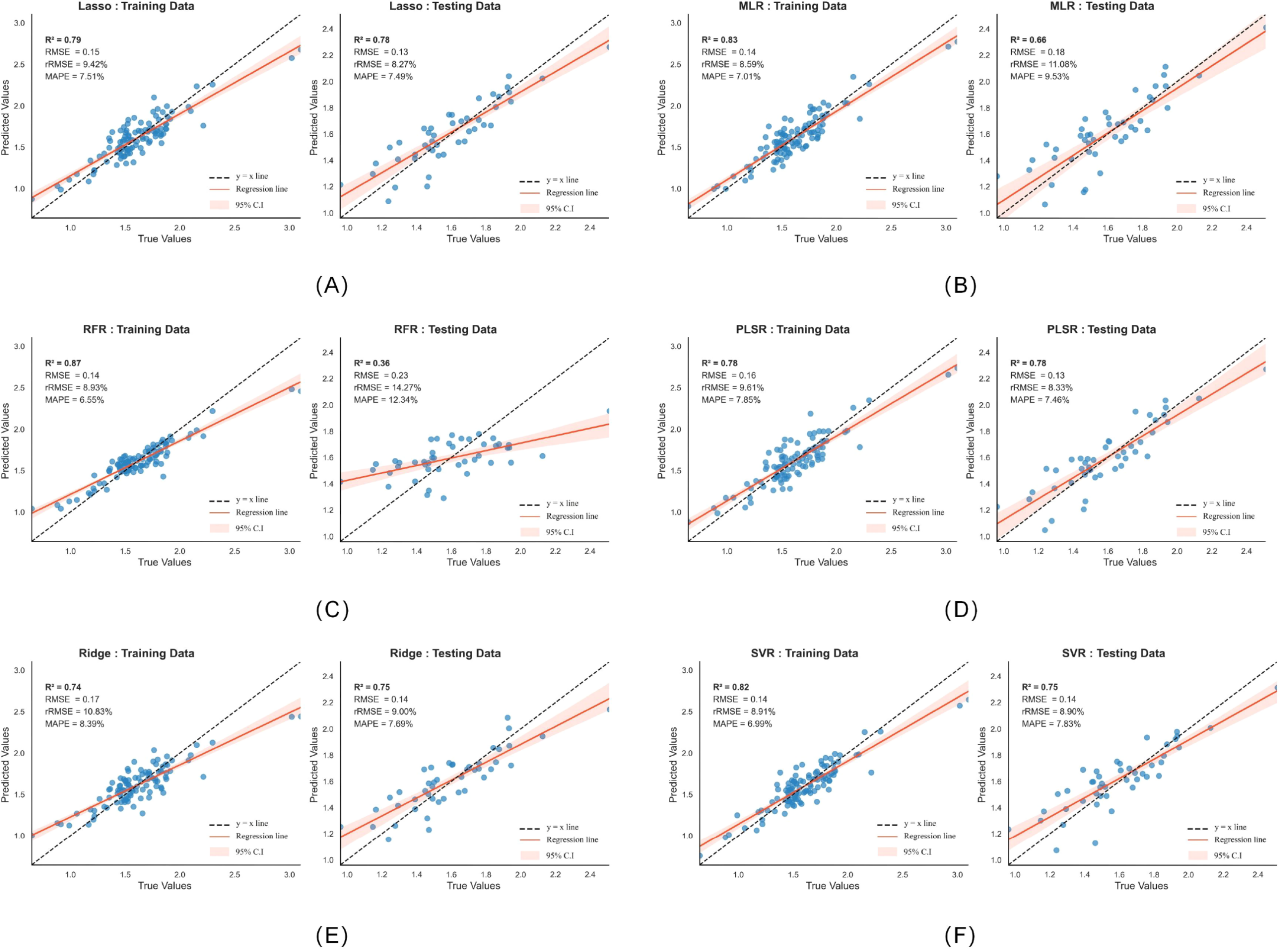
**(A)** Lasso model results; **(B)** MLR model results; **(C)** PLSR model results; **(D)** RFR model results; **(E)** Ridge model results; **(F)** SVR model results.

In the construction of the cotton canopy LCC inversion model, the six selected color features were used as input variables, with measured cotton LCC as the output. Six machine learning algorithms—LASSO, MLR, PLSR, RFR, Ridge, and SVR—were employed to build individual models. By dividing the dataset into training and testing sets, the models were developed using the training data and their predictive performance was evaluated on the testing set. The inversion performance of each model is illustrated in **Figure 9**.

This study compared the inversion performance of 6 models: Lasso, MLR, PLSR, RFR, Ridge, and SVR. The results are presented in **Table 6**. On the training set, RFR, MLR, and SVR demonstrated strong fitting capabilities, with R² values all exceeding 0.82 and RMSE values at the lowest level (0.14 mg/g). Their rRMSE and MAPE metrics were also the best among all models. Notably, RFR achieved the highest training set R² (0.87), indicating an exceptionally strong ability to fit the sample data.

**Table 6:** Comparison of 6 model parameters.

Model	Training				Testing			
	R2	RMSE	rRMSE	MAPE	R2	RMSE	rRMSE	MAPE
Lasso	0.79	0.15	9.42%	7.51%	0.78	0.13	8.27%	7.49%
MLR	0.83	0.14	8.59%	7.01%	0.66	0.18	11.08%	9.53%
PLSR	0.78	0.16	9.61%	7.85%	0.78	0.13	8.33%	7.46%
RFR	0.87	0.14	8.93%	6.55%	0.36	0.23	14.27%	12.34%
Ridge	0.74	0.17	10.83%	8.39%	0.75	0.14	9.00%	7.69%
SVR	0.82	0.14	8.91%	6.99%	0.75	0.14	8.90%	7.83%

However, a clear stratification emerged in the generalization performance of the models on the test set. RFR exhibited severe overfitting, with its R² sharply dropping to 0.36, accompanied by significantly worse rRMSE and MAPE values, indicating insufficient generalization ability for this task. MLR also showed a notable decline in performance, with its test set R² decreasing to 0.66, reflecting limited stability.

In contrast, SVR, Lasso, and PLSR demonstrated better generalization stability. Among them, SVR delivered the most balanced performance, with training and test set R² values of 0.82 and 0.75, respectively, and an RMSE consistently maintained at 0.14 mg/g. Other metrics showed minimal fluctuations, indicating its effective balance between fitting and generalization. Lasso and PLSR also exhibited good robustness, with test set R² values of 0.78 and slight improvements in some metrics compared to the training set, further validating their effectiveness in mitigating overfitting. Ridge regression performed stably but showed slightly inferior overall performance compared to SVR.

Based on the comparative analysis, the SVR model demonstrated the best overall performance within the 150-sample dataset.

To objectively evaluate the performance of each machine learning model in cotton canopy LCC inversion, model comparison and selection were conducted based on the 150 samples using a strategy of 10 repeated random splits (70% training, 30% testing) combined with 5-fold cross-validation tuning. The average performance metrics of each model across the 10 repetitions are presented in **Table 7**.

**Table 7:** Performance comparison of six machine learning models on the testing set (mean ± standard deviation).

Model	R²	RMSE (mg/g)	rRMSE (%)	MAPE (%)
LASSO	0.76 ± 0.03	0.14 ± 0.02	8.9 ± 1.2	7.8 ± 1.1
MLR	0.64 ± 0.06	0.18 ± 0.03	11.5 ± 1.8	10.2 ± 1.6
PLSR	0.75 ± 0.03	0.15 ± 0.02	9.2 ± 1.3	8.1 ± 1.2
RFR	0.36 ± 0.08	0.24 ± 0.04	15.3 ± 2.5	13.8 ± 2.3
Ridge	0.72 ± 0.03	0.16 ± 0.02	9.8 ± 1.4	8.6 ± 1.3
SVR	0.79 ± 0.03	0.14 ± 0.01	8.7 ± 0.9	7.6 ± 0.8

**Table 7** presents the performance of the six machine learning models across 10 repeated random splits. All metrics are reported as “mean ± standard deviation” to reflect the stability of model performance. It can be observed that the SVR model achieved the highest mean R² (0.79), the lowest RMSE (0.14 mg/g), and the smallest MAPE (7.6%) on the testing set, with relatively small standard deviations for all metrics. This indicates that the SVR model not only possesses high prediction accuracy but also exhibits stable performance with low sensitivity to data partitioning. In contrast, the RFR model achieved a testing R² of only 0.36, with the highest RMSE and MAPE values and large standard deviations, demonstrating typical overfitting characteristics and instability.

Based on the above comparison, the SVR model was identified as the best-performing model in this study, achieving a mean R² of 0.79 ± 0.03, RMSE of 0.14 ± 0.01 mg/g, and MAPE of 7.6% ± 0.8% on the testing set.

To further validate the robustness of the SVR model and exclude the potential influence of data interpolation methods on performance evaluation, a supplementary validation was conducted using the 60 original samples. The SVR model was retrained using a single random split (70% training, 30% testing) with the same color features and optimal hyperparameters as previously described. The validation results are presented in **Table 8**, showing that the model achieved an R² of 0.81 and an RMSE of 0.14 mg/g on the testing set, which is highly consistent with the 10-repetition results obtained from the 150 samples. This result strongly demonstrates that the LCC inversion method based on UAV RGB imagery and the SVR model possesses inherent effectiveness and robustness, and its excellent performance does not rely on EBK interpolation or specific data partitioning methods.

**Table 8:** Original sample model parameters.

Dataset	R2	RMSE	rRMSE	MAPE
Training	0.88	0.15	8.55%	6.52%
Testing	0.79	0.14	8.76%	7.13%

**Figure 10:**
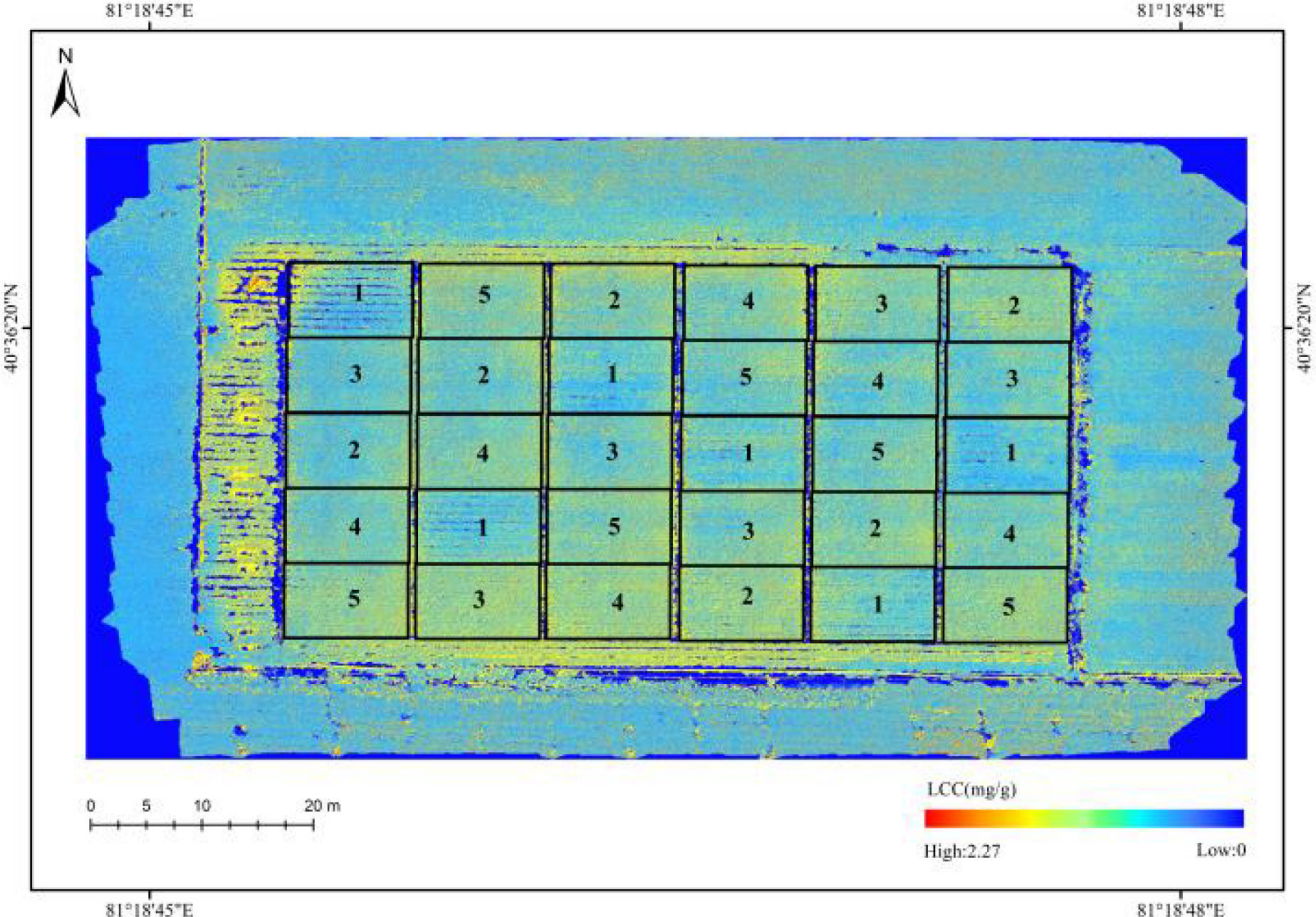
Chlorophyll content distribution map of cotton canopy in fields.

To investigate the spatial distribution of LCC in the cotton canopy, the R, G, and B components of each pixel in the field cotton image were first extracted, and their corresponding color feature values were calculated. These feature values were then input into the trained SVR model to invert the cotton canopy LCC pixel by pixel, generating a grayscale LCC distribution map. To enhance visualization, pseudocolor processing was applied to the grayscale image, resulting in a clear pseudocolor map that reflects the spatial distribution of chlorophyll in the cotton canopy, as shown in **Figure 10**.

In **Figure 10**, varying colors and their shades represent the spatial distribution differences of LCC in the cotton canopy. The dark blue areas correspond to road edges, soil, and non-image regions, where chlorophyll content is close to zero. Light blue and green areas indicate low LCC levels, ranging from 0.5 to 1.5 mg/g. Yellow to red regions represent higher LCC values, distributed between 1.5 and 2.27 mg/g. According to the experimental design, Treatment 1 served as the blank control group with no nitrogen fertilizer application. Since nitrogen is closely linked to chlorophyll synthesis, this treatment resulted in the lowest nitrogen uptake and consequently the lowest LCC in cotton. As shown in **Figure 10**, in the field cotton canopy LCC distribution map, the cotton LCC under this treatment was also the lowest among all five treatments across repeated experiments, which aligns with the expectations of the experimental design. The field cotton canopy LCC distribution map, generated based on the SVR model inversion results, clearly illustrates the spatial variability characteristics of LCC in the field cotton canopy. It provides a visual representation of the nitrogen nutritional status and overall growth condition of the cotton plants, offering scientific support for precision field management.

## 4 Discussion

Chlorophyll, as the core pigment in plant photosynthesis, exhibits significant influences on both the physiological state of leaves and their spectral reflectance characteristics, with its content variations playing a crucial role (Demotes-Mainard et al., 2008; Xu et al., 2019; Sonobe et al., 2020). There exists a significant correlation between vegetation LCC and color features. Therefore, utilizing R, G, and B color components to monitor vegetation growth status has emerged as a rapid, economical, and technically feasible method suitable for small-scale farmland applications. This approach also lays the foundation for UAV remote sensing estimation of cotton canopy LCC.

Through field experiments, this study cultivated 2 cotton varieties and established 5 nitrogen application gradients to ensure the broad adaptability of the constructed models. The introduction of RTK technology significantly enhanced the positional accuracy of leaf samples in UAV images, providing a reliable foundation for subsequent image segmentation and feature extraction. During the model construction phase, a systematic comparison of 6 machine learning algorithms revealed that the SVR model excelled in handling nonlinear relationships, with its predictions showing high consistency with measured data. The spatial distribution map of cotton canopy LCC generated based on this model clearly reflects intra-field distribution differences, offering scientific support for precision fertilization. Specifically, this study leveraged RTK high-precision positioning technology to accurately locate cotton leaf samples, screened color features highly correlated with LCC through correlation analysis, and achieved high-precision LCC inversion using the SVR model. The outstanding performance of the SVR model can be attributed to its ability to capture both linear and nonlinear relationships while maintaining strong generalization capabilities even under small sample conditions (Feng et al., 2020; Fei et al., 2023). Consequently, despite the limited sample size in this study, the model demonstrated stable predictive performance.

Regarding the data strategy, 150 EBK-interpolated samples were employed as the primary modeling dataset in this study. The rationale for this strategy lies in the ability of EBK interpolation to provide a more stable data foundation for model comparison. Although the 60 original samples were reliable, their limited size made them susceptible to the influence of individual outliers. The 150 interpolated samples smoothed local stochastic variability (with the standard deviation decreasing from 0.46 mg/g to 0.32 mg/g), enabling the six machine learning algorithms to be fairly compared on a relatively stable dataset, thereby more accurately reflecting the inherent learning capabilities of each model.

To validate the soundness of this strategy, an independent validation was conducted using the 60 original samples to test the optimal SVR model constructed from the 150 samples. The results showed that the model achieved excellent performance on the 60 original samples, with an R² of 0.81 and an RMSE of 0.14 mg/g, which was highly consistent with the internal validation results based on the 150 samples (R² = 0.79 ± 0.03, RMSE = 0.14 ± 0.01 mg/g). This comparison is significant: if the interpolation process had introduced spurious information or artificially exaggerated data patterns, the validation results on the original samples would have been substantially lower than the internal validation results—a phenomenon not observed in this study. This strongly demonstrates that the SVR model constructed from the 150 samples possesses inherent effectiveness and robustness, and its excellent performance does not depend on the introduction of interpolated data.

Previous studies have largely relied on MS and HS UAVs for crop chlorophyll detection, achieving relatively high accuracy. For example, Narmilan et al. (2022) utilized MS UAVs to detect LCC in sugarcane, where the XGBoost model achieved an R² of 0.98 and an RMSE of 0.78 after feature selection. Similarly, An et al. (2020) predicted rice LCC based on HS UAVs, with the RFR model demonstrating strong predictive capabilities by achieving R² values of 0.95 and 0.80 on the training and test sets, respectively. These results highlight the significant advantages of MS and HS UAVs in chlorophyll detection. However, Qiao et al. (2022) reported a training set R² of 0.753 when using MS UAV to monitor maize canopy chlorophyll, which is slightly lower than the R² values obtained in this study.

The improvement in accuracy in this study can be primarily attributed to the adopted RTK high-precision spatial alignment technology. Unlike previous studies, which often relied on coarse matching between sampling points and imagery, this research achieved pixel-level precise matching between leaf samples and canopy images through RTK technology. This approach fundamentally ensures that the image features used for modeling accurately reflect the biochemical parameters of the corresponding leaves. By minimizing positional deviations at the data level, it establishes a solid foundation for high-precision inversion. It is this technological pathway that ultimately enables low-cost RGB UAV to achieve LCC inversion accuracy comparable to that of HS and MS systems.

Although this study has achieved high-precision LCC inversion based on RGB UAVs, certain limitations remain, which point to potential directions for future research.

Firstly, the model inputs in this study rely on manually selected color features. To overcome the limitations of traditional feature engineering, future work could explore the use of deep learning models, such as convolutional neural networks (CNNs), to directly learn deep-level texture and spatial features related to LCC from raw RGB imagery. This approach has the potential to further enhance model accuracy and robustness while maintaining the advantage of low cost.

Secondly, the data in this study were collected from a single growth stage of cotton (the bud stage). To improve the generalizability of the model across different growth stages, subsequent research should conduct dynamic monitoring throughout the entire growth period. This would involve systematically analyzing how the relationship between color features and LCC evolves with phenological stages (Adão et al., 2017) and developing segmented inversion models or temporal normalization methods tailored to different growth phases.

Lastly, the model in this study was developed and validated exclusively in the Alar cotton-growing region. To assess the transferability and general applicability of the method, future research should aim to extensively validate it across a broader range of crop types (e.g., corn and wheat) and ecological regions. This would help promote the large-scale application of this low-cost technical solution in smart agriculture.

In summary, although LCC inversion based on RGB UAVs still has certain limitations in terms of accuracy, its low-cost advantage demonstrates broad application prospects for field crop monitoring. By integrating strategies such as multi-temporal analysis, deep learning, and validation across multiple crop types, it is possible to gradually narrow the performance gap with professional-grade remote sensing equipment in physiological parameter inversion. This, in turn, will provide robust technical support for the real-time, precise, and large-scale monitoring of crop growth status.

Most importantly, this study has the potential to resolve the long-standing dilemma in the Alar region regarding cotton field chlorophyll detection—the trade-off between “accuracy without economy” and “economy without accuracy.” The low-cost RGB UAV enables precise LCC detection while transforming the traditional “experience-based fertilization” model. The generated LCC distribution maps can intuitively guide variable-rate fertilization, with an estimated reduction in nitrogen fertilizer application of 15-20%, thereby balancing yield maintenance with environmental protection.

## 5 Conclusions

Based on water and fertilizer management in cotton fields, this study utilized a UAV equipped with an RGB camera to collect cotton canopy images. Through RTK high-precision positioning, image processing, correlation analysis, and machine learning, the inversion of cotton canopy LCC was achieved. The main conclusions are as follows:

1. The SVR model demonstrated training and test set R² values of 0.82 and 0.75, RMSE values of 0.14 mg/g and 0.14 mg/g, rRMSE values of 8.91% and 8.90%, and MAPE values of 6.99% and 7.83%, respectively. Its overall performance was superior to that of the Lasso, MLR, PLSR, RFR, and Ridge models.
2. Applying the SVR model to pixel-level LCC inversion in small-scale cotton canopy imagery, combined with pseudocolor imaging technology, can intuitively reveal the spatial heterogeneity characteristics of LCC in cotton fields. This provides an effective approach for monitoring cotton growth status.
3. By achieving high-precision matching between RTK positioning information and cotton canopy imagery, this study ensured spatial consistency at the data level and significantly improved the accuracy of the cotton field LCC prediction model. This provides an important methodological reference for crop physiological parameter inversion based on low-cost remote sensing platforms.

## Data Availability

The original contributions presented in the study are included in the article/supplementary material. Further inquiries can be directed to the corresponding authors.
